# Tuition reduction is the key factor determining tax burden of graduate students under the Tax Cuts and Job Act

**DOI:** 10.12688/f1000research.13385.2

**Published:** 2018-02-05

**Authors:** Patricia M. Lawston, Michael T. Parker

**Affiliations:** 1 Earth System Science Interdisciplinary Center, University of Maryland, College Park, MD, USA; 2 Hydrological Sciences Laboratory, NASA Goddard Space Flight Center, Greenbelt, MD, USA; 3 Department of Immunobiology, Yale University School of Medicine, New Haven, CT, USA

**Keywords:** higher education, graduate students, tax reform, taxes, tuition reduction

## Abstract

**Background**: The proposed Tax Cuts and Jobs Act (H.R.1) has stirred significant public debate on the future of American economics.  While supporters of the plan have championed it as a necessity for economic revitalization, detractors have pointed out areas of serious concern, particularly for low- and middle-income Americans.  One particularly alarming facet of the plan is the radical change to education finance programs and taxation of students in higher education.

**Methods**:  By analyzing actual income and tuition of a public and a private university student, as well as the ‘average’ graduate student, we investigated the effect of both the House and Senate versions of H.R. 1 on taxation of students of various family structures.

**Results**:  Our findings indicate that taxable tuition would be the greatest contributor to graduate student tax burden across all four categories of filing status.  However, when tuition reduction is upheld or a student is on sustaining fees rather than full tuition, graduate students would realize decreases in taxation.

**Conclusions**:  Overall, we conclude that removal of tuition reduction would result in enormous tax burdens for graduate students and their families and that these effects are dependent not only on the status of the student in their degree program but also on their tuition and stipend, and therefore the institution they attend.

## Introduction

As the Tax Cuts and Jobs Act (H.R.1) inches nearer to becoming law, graduate student concern is mounting to an all-time high. This bill contains changes to how various facets of education are taxed, including student loan interest, education assistance, tax credits, and debt forgiveness ^1^. However, the most jarring change for graduate students is the removal of tuition reduction ^2^, the provision in current tax code allowing students to exclude money provided by their graduate program to cover their tuition costs from their taxable income. Students are self-reporting that this would increase their yearly taxes between two- and four-fold, resulting in effective tax rates upwards of 33% ^1, 3– 7^. However, self-reporting can be misleading without in-depth tax calculations included for verification. To address these issues and to help graduate students and elected officials better inform themselves on the impact of proposed tax reform, we analyzed the historical taxes of two graduate students, one public university student and one private university student (the authors of this work), over the course of four years (2013–2016) in science PhD programs. We then extrapolated these analyses for family structures typical of graduate students, including relevant historical and proposed tax deductions and exemptions.

## Methods

**Tax calculation notes:** Because the Senate version of the Tax Cuts and Jobs act is not yet finalized, we used the “Chairman’s Mark” tax brackets for our calculations ^8^. We excluded the Additional Child Tax Credit from our analyses because this requires more nuanced calculation of tax before it can be determined and is therefore more complex than is appropriate for this simplified, generic example ^9^.

**Total taxes owed (TTO)**: For each year 2013–2016, the appropriate tax brackets were applied to calculate the total taxes owed (TTO) based on stipends, health waivers, and tuition ^10– 17^. Representation of TTO is calculated by year, and differences in TTO for each proposed tax structure are in relation to that same year’s historical tax structure TTO calculation. The income values used for these calculations as well as the numerous factors considered in tax calculations (i.e., column headers) can all be found in Datasets 1–3.

**Effective tax rate (ETR)**: Calculations of TTO were made as described above. Then, the TTO was divided by the stipend (not health waiver and tuition) to calculate the effective tax rate (ETR). Differences in ETR for each proposed tax structure are in relation to that same year’s historical tax structure ETR.

**Figure 1.**
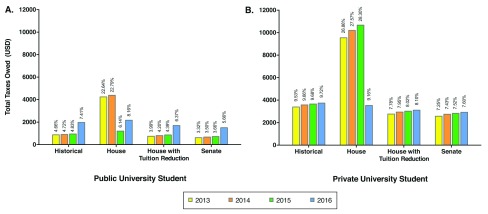
Effect of proposed tax plans on total taxes owed by single graduate students using tax data from 2013–2016. Total Taxes Owed (TTO) represented in USD for single filing public ( **A**) and private ( **B**) university graduate students. TTO for each year (2013–2016) are color coded by year and grouped according to the tax structure utilized to make each calculation ( Table 1). These structures were the historical taxation structures for years 2013–2016, the proposed House tax plan, the proposed House plan with Tuition Reduction, and the proposed Senate tax plan. Above each bar is the calculated Effective Tax Rate (ETR) for each scenario, represented as a percent of income.

**Figure 2.**
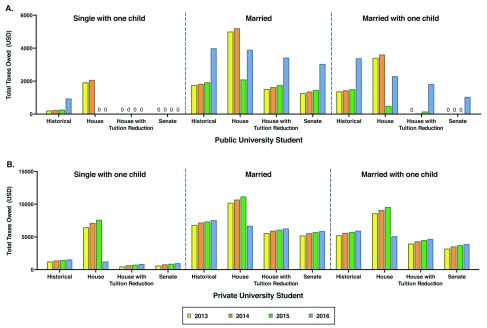
Effect of proposed tax plans on total taxes owed by graduate student taxpayers with children and/or spouses filing jointly using tax data from 2013–2016. Total Taxes Owed (TTO) represented in USD for public ( **A**) and private ( **B**) university graduate students filing either as single with one child, married (filing jointly), or married with one child (filing jointly). TTO for each year (2013–2016) are color coded by year and grouped according to the tax structure utilized to make each calculation ( Table 2). These structures were the historical taxation structures for years 2013–2016, the proposed House tax plan, the proposed House plan with Tuition Reduction, and the proposed Senate tax plan. A “0” represents no taxes owed.

**Sustaining fees**: Once graduate students progress from the class and teaching portions of their graduate work into the research intensive/exclusive portion, many schools no longer charge full tuition, but rather a smaller fee termed a “sustaining fee”. In the case of both graduate student salaries utilized in this work, this was the case, and the sustaining fees for each can be found in Datasets 1 and 2. The public student was on sustaining status in 2015 and 2016 while the private student was on sustaining status in 2016. The reduction in taxation is drastic when students transition from tuition status to sustaining status under plans that do not contain the tuition reduction provision (see Figure 1 and Figure 2).

**Estimating the ‘average’ graduate student**: We estimated the ‘average’ stipend using the current Glassdoor approximation of $30,603 ^18^ and then back-calculated for 2013–2015 assuming a 2% yearly increase, as was observed in the public and private student stipends. One consideration in this approximation is that these data are self-reported and not field specified and since STEM fields typically have higher stipends than humanities, bias in either discipline’s direction may skew this value. Tuition was calculated using approximate average public graduate school tuition ($30,000) and average private graduate school tuition ($40,000) ^19^. Then, we weighted the proportion based on an American Academy of Sciences approximation that 60% of PhDs are awarded from public universities ^20^. This gave an average tuition across all students of $34,000 for 2016, which was then back-calculated for 2013–2015 assuming a 2% yearly increase. For health waivers, there is currently no conglomeration of public data available of the average worth provided by universities, so the value used was the average of the public and private student’s actual health waivers ($1,500).

**Cumulative differential tax burden (CDTB)**: This value was calculated using the sum of TTO for 2013–2016 under each proposed tax plan (House, House with Tuition Reduction, Senate) minus the sum of TTO for 2013–2016 under historical tax structure. The result is four-year TTO for each filing status (single, single with one child, married, married with one child) under each proposed tax plan.

## Results

Single individuals represent the largest category of graduate students (approximately 44.41% ^21^). Table 1 exhibits the calculated taxes for the period 2013–2016 for one individual each from a public and private university using historical tax structure ^10– 15^, the House proposed plan ^16^, and the Senate proposed plan ^8, 17^. These calculations were made using income, tuition, health waivers, the standard deduction (typical for graduate students), and deductions and exemptions that are germane to this group. Representation of the total taxes owed (TTO) and effective tax rates (ETR = TTO / stipend) are shown in Table 1, showing maximum increases under the House plan of approximately +386% and +19%, respectively. For the public student, this jump in both categories can partly be attributed to a low initial tax burden that is substantially increased when tuition is considered taxable income. The increases are more modest in years where this student was on sustaining fees (2015 and 2016) rather than full tuition. When considering the Senate plan, which does not tax tuition ^22^, there are actually large decreases in TTO and modest decreases in ETR (maximum of approximately -29% and -1.75%, respectively). Interestingly, when considering the House plan without taxable tuition, the TTO and ETR both decrease as well (maximum of approximately -14.5% and -1.0%, respectively). This highlights the extreme effect this one change would have on graduate student tax burden.

**Table 1.** Calculation of 2013–2016 taxes for single, graduate student taxpayers using historical and proposed tax plans.

	Public Student (Single)				Private Student (Single)			
Total Taxes Owed (TTO)	2013	2014	2015 *	2016 *	2013	2014	2015	2016 *
Historical	$872.45	$907.23	$952.09	$1,976.25	$3,387.75	$3,573.75	$3,648.75	$3,736.55
House	$4,238.34	$4,380.16	$1,211.67	$2,175.32	$9,550.13	$10,200.63	$10,669.13	$3,523.68
House w/Reduction	$746.94	$806.68	$866.67	$1,700.00	$2,767.20	$2,940.00	$3,024.00	$3,114.00
Senate	$622.45	$672.23	$722.23	$1,509.50	$2,576.70	$2,749.50	$2,833.50	$2,923.50

Effective Tax Rate (ETR)	2013	2014	2015 *	2016 *	2013	2014	2015	2016 *
Historical	4.66%	4.72%	4.83%	7.41%	9.53%	9.66%	9.68%	9.72%
House	22.64%	22.79%	6.14%	8.16%	26.86%	27.57%	28.30%	9.16%
House w/Reduction	3.99%	4.20%	4.39%	6.37%	7.78%	7.95%	8.02%	8.10%
Senate	3.32%	3.50%	3.66%	5.66%	7.25%	7.43%	7.52%	7.60%

% Change from Historical TTO	2013	2014	2015 *	2016 *	2013	2014	2015	2016 *
House	385.80%	382.80%	27.26%	10.07%	181.90%	185.43%	192.41%	-5.70%
House w/Reduction	-14.39%	-11.08%	-8.97%	-13.98%	-18.32%	-17.73%	-17.12%	-16.66%
Senate	-28.66%	-25.90%	-24.14%	-23.62%	-23.94%	-23.06%	-22.34%	-21.76%

Difference from Historical ETR	2013	2014	2015 *	2016 *	2013	2014	2015	2016 *
House	17.98%	18.07%	1.32%	0.75%	17.33%	17.91%	18.62%	-0.55%
House w/Reduction	-0.67%	-0.52%	-0.43%	-1.04%	-1.75%	-1.71%	-1.66%	-1.62%
Senate	-1.34%	-1.22%	-1.17%	-1.75%	-2.28%	-2.23%	-2.16%	-2.11%

*student on sustaining status, see Datasets 1–3

The results for the student from a private university are similar to those above ( Table 1). The House plan results in approximate maximum increases of +192% TTO and +19% ETR, while both the House plan that retains tuition reduction and the Senate plan result in decreased TTO and ETR (approximate maximum of -18.5% TTO and -1.75% ETR for the House plan without taxable tuition; -24% TTO and -2.5% ETR for Senate plan). This student also went from full tuition to sustaining between 2015 and 2016, explaining the modest decrease in TTO and ETR.

Figure 1 shows, in U.S. Dollars (USD), the TTO for single individuals from a public (A) and a private (B) university based upon the type of tax plan applied. For the public student, TTO increases under the House plan, and decreases under the House plan with tuition reduction and the Senate plan ( Figure 1A). When tuition is taxed and the student is on full tuition, the TTO increases from $872 to $4238 in 2013 and from $907 to $4380 in 2014. For the private school student, who was on full tuition from 2013–2015, the increases in TTO under the House plan are $3,388 to $9,550 in 2013, $3,574 to $10,201 in 2014, and $3,649 to $10,669 in 2015 ( Figure 1B). Also, similar to the public student calculations, when the private student transitioned to sustaining fees in 2016, they would realize a decreased TTO under the House plan (-$213), although this decrease would be even greater without taxable tuition and with the Senate plan (-$623 and -$813, respectively). Overall, these analyses indicate that the proposed tax plans would generally decrease a single graduate student’s tax burden unless tuition is treated as taxable income, in which case there would be huge increases in TTO.

The changing status and income of graduate students over their programs and the effect this has on taxation is worth noting. Students go on and off of fellowships, affecting the amount and duration of their pay. The public student’s stipend was guaranteed for nine months of the year, but when funded by an outside (e.g., NSF) fellowship, the stipend was year-long, leading to greater annual income when on fellowship. And in later years of their degree, many students no longer have traditional tuition costs, but rather pay sustaining fees, greatly reducing their effective income under the House plan. In Figure 1A, the TTO of the public student responds to these fluctuating statuses under each plan. In 2013 and 2014, this student was off of fellowship and on full tuition, then went off of full tuition for 2015 and 2016, and in 2016 was on fellowship. Between 2014 and 2015, the transition to sustaining fees has little effect on plans that do not tax tuition, but under the House plan there is a large decrease (-$3,168), though this plan still results in an increase as compared to the others (+$260 vs. historical taxation). This highlights that sustaining fee status greatly reduces the effect of the House tax plan on graduate student tax increases. When the student goes onto fellowship in 2016 their stipend increases, with a concomitant increase in taxation. However, the increase in taxes under the House plan vs. the historical structure remains low (+$199), exhibiting that an increase in stipend (~30% in this case) has minimal effect on taxation as compared to that of taxing tuition.

While the above data represent the major population of graduate students, they do not paint the whole picture of tax scenarios as a graduate student. We identified three other common family structures of graduate students and calculated taxes for each based on historical and proposed tax policies. These groups and their proportion of the graduate student population were: Single with dependents (12.30%), married without dependents (14.95%), and married with dependents (28.34%) ^21^. It is important to mention that in keeping with the subject of this study, namely, graduate students, married couple calculations assume that both partners are students at the same university, and therefore the income, tuition, and health waivers are double that of a single student. Another factor that we controlled for simplicity of calculations was to assume that families that have children only had one.

Table 2 contains the tax calculations for each of the abovementioned groups using historical and proposed tax structures. Similar to single filers, the House plan would increase graduate student taxes across the board when tuition is considered as taxable. For the public student, TTO and ETR would increase an approximate maximum of +866% / +9.5% as a single filer with one child, +188% / +8.8% as a married couple filing jointly, and +385% / +5.7% as a married couple filing jointly with one child. In all cases, when tuition is not taxed in proposed plans, decreases in TTO and ETR are observed as compared to historical tax structures. Approximate maximum decreases in TTO and ETR under the Senate plan are -29% / -1.8% for Married Filing Jointly (MFJ) and -29% / -4.5% for MFJ with one child. The House plan without taxable tuition has similar, albeit more modest decreases in TTO and ETR (MFJ = -14.5% / -1.1%; MFJ with one child = -14.5% / -3.7%). Strikingly, the single filer with one child would actually owe no taxes to the government for any year if tuition is not taxable.

**Table 2.** Calculation of 2013–2016 taxes for graduate student taxpayers with children and/or spouses filing jointly using historical and proposed tax plans.

Single + Child								
	Public				Private			
Total Taxes Owed (TTO)	2013	2014	2015 *	2016 *	2013	2014	2015	2016 *
Historical	$197.45	$222.23	$247.23	$926.66	$1,184.00	$1,352.50	$1,410.00	$1,495.00
House	$1,906.34	$2,048.16	$0.00	$0.00	$6,425.13	$7,075.63	$7,544.13	$1,191.68
House w/Reduction	$0.00	$0.00	$0.00	$0.00	$435.20	$608.00	$692.00	$782.00
Senate	$0.00	$0.00	$0.00	$0.00	$576.70	$749.50	$833.50	$923.50

Effective Tax Rate (ETR)	2013	2014	2015 *	2016 *	2013	2014	2015	2016 *
Historical	1.05%	1.16%	1.25%	3.47%	3.33%	3.66%	3.74%	3.89%
House	10.18%	10.66%	0.00%	0.00%	18.07%	19.12%	20.01%	3.10%
House w/Reduction	0.00%	0.00%	0.00%	0.00%	1.22%	1.64%	1.84%	2.03%
Senate	0.00%	0.00%	0.00%	0.00%	1.62%	2.03%	2.21%	2.40%

% Change from Historical TTO	2013	2014	2015 *	2016 *	2013	2014	2015	2016 *
House	865.49%	821.63%	-100.00%	-100.00%	442.66%	423.15%	435.04%	-20.29%
House w/Reduction	-100.00%	-100.00%	-100.00%	-100.00%	-63.24%	-55.05%	-50.92%	-47.69%
Senate	-100.00%	-100.00%	-100.00%	-100.00%	-51.29%	-44.58%	-40.89%	-38.23%

Difference from Historical ETR	2013	2014	2015 *	2016 *	2013	2014	2015	2016 *
House	9.13%	9.50%	-1.25%	-3.47%	14.74%	15.47%	16.27%	-0.79%
House w/Reduction	-1.05%	-1.16%	-1.25%	-3.47%	-2.11%	-2.01%	-1.90%	-1.85%
Senate	-1.05%	-1.16%	-1.25%	-3.47%	-1.71%	-1.63%	-1.53%	-1.49%

*student on sustaining status, see Datasets 1–3

**Table 2 continued.** Calculation of 2013–2016 taxes for graduate student taxpayers with children and/or spouses filing jointly using historical and proposed tax plans.

Married Filing Jointly								
	Public				Private			
Total Taxes Owed (TTO)	2013	2014	2015 *	2016 *	2013	2014	2015	2016 *
Historical	$1,744.90	$1,814.46	$1,904.18	$3,967.49	$6,775.50	$7,147.50	$7,297.50	$7,502.50
House	$4,985.28	$5,186.84	$2,078.35	$3,875.31	$10,159.20	$10,644.24	$11,119.00	$6,637.68
House w/Reduction	$1,493.88	$1,613.36	$1,733.35	$3,399.99	$5,534.40	$5,880.00	$6,048.00	$6,228.00
Senate	$1,244.90	$1,344.46	$1,444.46	$3,018.99	$5,153.40	$5,499.00	$5,667.00	$5,847.00

Effective Tax Rate (ETR)	2013	2014	2015 *	2016 *	2013	2014	2015	2016 *
Historical	4.66%	4.72%	4.83%	7.44%	9.53%	9.66%	9.68%	9.76%
House	13.31%	13.49%	5.27%	7.27%	14.28%	14.38%	14.75%	8.63%
House w/Reduction	3.99%	4.20%	4.39%	6.37%	7.78%	7.95%	8.02%	8.10%
Senate	3.32%	3.50%	3.66%	5.66%	7.25%	7.43%	7.52%	7.60%

% Change from Historical TTO	2013	2014	2015 *	2016 *	2013	2014	2015	2016 *
House	185.71%	185.86%	9.15%	-2.32%	49.94%	48.92%	52.37%	-11.53%
House w/Reduction	-14.39%	-11.08%	-8.97%	-14.30%	-18.32%	-17.73%	-17.12%	-16.99%
Senate	-28.66%	-25.90%	-24.14%	-23.91%	-23.94%	-23.06%	-22.34%	-22.07%

Difference from Historical ETR	2013	2014	2015 *	2016 *	2013	2014	2015	2016 *
House	8.65%	8.77%	0.44%	-0.17%	4.76%	4.73%	5.07%	-1.12%
House w/Reduction	-0.67%	-0.52%	-0.43%	-1.06%	-1.75%	-1.71%	-1.66%	-1.66%
Senate	-1.34%	-1.22%	-1.17%	-1.78%	-2.28%	-2.23%	-2.16%	-2.15%

*student on sustaining status, see Datasets 1–3

**Table 2 continued.** Calculation of 2013–2016 taxes for graduate student taxpayers with children and/or spouses filing jointly using historical and proposed tax plans.

Married Filing Jointly + Child								
	Public				Private			
Total Taxes Owed (TTO)	2013	2014	2015 *	2016 *	2013	2014	2015	2016 *
Historical	$1,354.90	$1,419.46	$1,484.46	$3,359.99	$5,190.50	$5,555.00	$5,697.50	$5,895.00
House	$3,385.28	$3,586.84	$478.35	$2,275.31	$8,559.20	$9,044.24	$9,519.00	$5,037.68
House w/Reduction	$0.00	$13.36	$133.35	$1,799.99	$3,934.40	$4,280.00	$4,448.00	$4,628.00
Senate	$0.00	$0.00	$0.00	$1,018.99	$3,153.40	$3,499.00	$3,667.00	$3,847.00

Effective Tax Rate (ETR)	2013	2014	2015 *	2016 *	2013	2014	2015	2016 *
Historical	3.62%	3.69%	3.76%	6.30%	7.30%	7.51%	7.56%	7.67%
House	9.04%	9.33%	1.21%	4.27%	12.03%	12.22%	12.62%	6.55%
House w/Reduction	0.00%	0.03%	0.34%	3.37%	5.53%	5.78%	5.90%	6.02%
Senate	0.00%	0.00%	0.00%	1.91%	4.43%	4.73%	4.86%	5.00%

% Change from Historical TTO	2013	2014	2015 *	2016 *	2013	2014	2015	2016 *
House	149.85%	152.69%	-67.78%	-32.28%	64.90%	62.81%	67.07%	-14.54%
House w/Reduction	-100.00%	-99.06%	-91.02%	-46.43%	-24.20%	-22.95%	-21.93%	-21.49%
Senate	-100.00%	-100.00%	-100.00%	-69.67%	-39.25%	-37.01%	-35.64%	-34.74%

Difference from Historical ETR	2013	2014	2015 *	2016 *	2013	2014	2015	2016 *
House	5.42%	5.64%	-2.55%	-2.03%	4.74%	4.72%	5.07%	-1.11%
House w/Reduction	-3.62%	-3.66%	-3.43%	-2.92%	-1.77%	-1.72%	-1.66%	-1.66%
Senate	-3.62%	-3.69%	-3.76%	-4.39%	-2.86%	-2.78%	-2.69%	-2.66%

*student on sustaining status, see Datasets 1–3

The private student would encounter similar tax scenarios, with approximate maximum increases in TTO and ETR under the House plan of +443% / +16.3% as a single with one child, +52.4% / +5.1% as MFJ, and +67.1% / +5.07% as MFJ with one child. Removal of taxable tuition results in decreased taxation in all cases, with the House plan performing better for singles with one child (-63.25% / -2.15%) and the Senate plan performing better for married filers (MFJ = -24% / -1.75%; MFJ with one child = -39.25% / -1.8%).

Figure 2 shows the range of TTO in USD for the various family structures of graduate students from a public (A) and a private (B) university based upon the type of tax plan applied. When tuition is taxed under the House plan, TTO increases in all cases for all filers. For the public student these increases were greatest with 2014 data (single with one child = $222 to 2,048; MFJ = $1,814 to $5,187; MFJ with one child = $1,419 to $3,587), the last year this student was on full tuition ( Figure 2A). When calculations were made using the House plan without taxable tuition and/or the Senate plan, TTO decreased in all cases. And when the student was no longer on full tuition (2015 and 2016) TTO actually decreased when comparing the House plan with the historical structure in all cases (with the exception of MFJ in 2015). In 2015, these changes were: Single = -$260; single with one child = -$247; MFJ = +$174; MFJ with one child = -$1,006. In 2016, these changes were: Single = -$199; single with one child = -$927; MFJ = -$92; MFJ with one child = -$1,085.

The increase in TTO was also largest for the private school student in the last year of full tuition, 2015 (single with one child = $1,410 to 7,544; MFJ = $7,298 to $11,119; MFJ with one child = $5,698 to $9,519) ( Figure 2B). For all family types and in all cases, when the private school student was no longer on full tuition or when calculations were made using proposed plans without taxable tuition, there were decreases in TTO, though these were most striking for couples (ex.: 2016, MFJ with one child: Historical = $5,895; House = $5,038, [-$857]; House without taxable tuition = $4,628, [-$1,267]; Senate = $3,847, [-$2,048]). Overall, these analyses indicate that the proposed tax plans would generally decrease graduate student family tax burden unless tuition is treated as taxable income. Both new plans benefit students in the form of decreased taxation when they are on sustaining fees near the end of their degrees. Additionally, the Senate plan results in more tax relief than any other tax structure for all family types.

**Figure 3.**
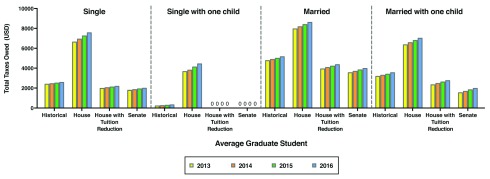
Effect of proposed tax plans on the average graduate student. Total Taxes Owed (TTO) represented in USD for an ‘average’ university graduate student filing either as single, single with one child, married (filing jointly), or married with one child (filing jointly). TTO for each year (2013–2016) are color coded by year and grouped according to the tax structure utilized to make each calculation ( Datasets 1–3, Tables S1–S6). These structures were the historical taxation structures for years 2013–2016, the proposed House tax plan, the proposed House plan with Tuition Reduction, and the proposed Senate tax plan. A “0” represents no taxes owed.

While utilizing taxes from representative students is useful to delineate between type of institution, it is more generally applicable to analyze the average income, tuition, and health waiver of a typical graduate student. These calculations ( Dataset 3) follow the same trends as those for the individuals above, and the effect of various tax plans on TTO are shown below in Figure 3. Increases in TTO under the House plan were most drastic using 2016 estimates (single = $2,566 to $7,550; single with one child = $320 to $4,425; MFJ = $5,148 to $8,604; MFJ with one child = $3,540 to $7,004). As before, removal of taxable tuition from the House plan and/or the Senate plan results in overall decreases in TTO (Example, 2016, single: Historical = $2,566; House without taxable tuition = $2,172, [-$394]; Senate = $1,982, [-$584]).

**Figure 4.**
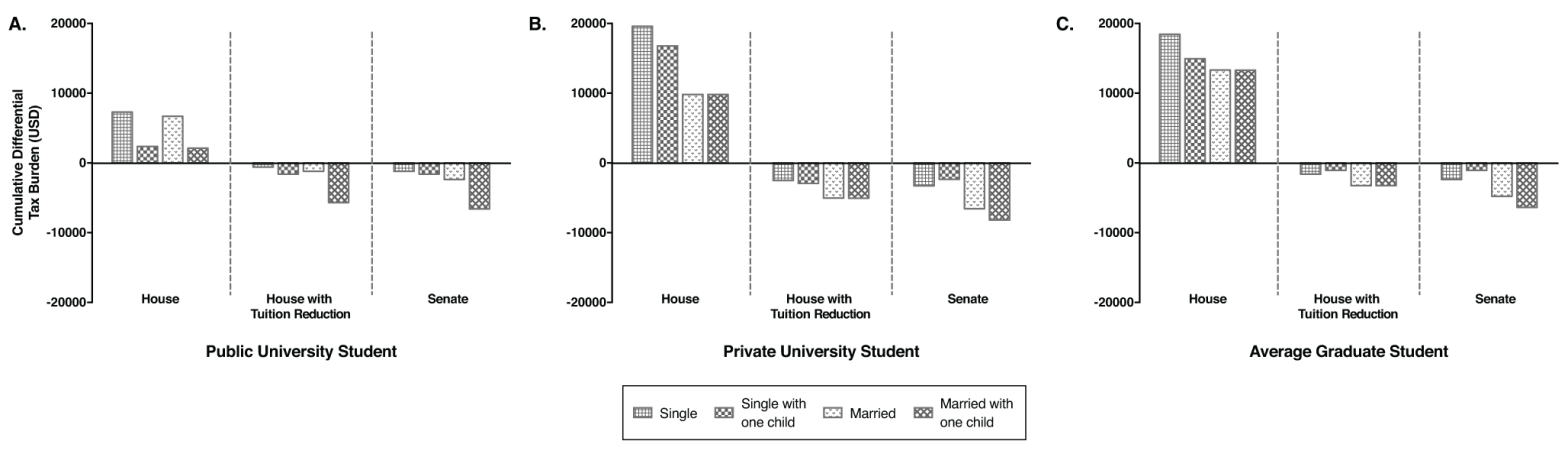
Four-year cumulative taxation for various family types under each tax structure. Cumulative Differential Tax Burden (CDTB) represented in USD for a public university ( **A**), private university ( **B**), or ‘average’ ( **C**) graduate student. CDTB is the TTO for four years (2013–2016) and is coded by filing status (single, single with one child, married [filing jointly], or married with one child [filing jointly]) and grouped according to the tax structure utilized to make each calculation ( Datasets 1–3, Tables S1–S6). These structures were the historical taxation structures for years 2013–2016, the proposed House tax plan, the proposed House plan with Tuition Reduction, and the proposed Senate tax plan.

The cumulative effect of tax reform on graduate students is likely the most informative measure of potential impacts. In Figure 4, we calculate the cumulative difference in taxes over four years with both former and proposed tax plans for the public student (A), private student (B), and average student (C). For the public student, the cumulative differential tax burden (CDTB, see Methods) over four years under the House tax plan is $7,297 for a single filer and $2,107 for a single with one child ( Figure 4A). These figures are marginally lower for married filers ($6,695 to $2,361), as expected. These results highlight that, under the House plan, dependent child status has a greater buffering impact than does marriage. For the private student, whose stipend and tuition are greater than the public student, the CDTB values are: Single = $19,597; single with one child = $16,795; MFJ = $9,837; MFJ with one child = $9,822 ( Figure 4B). For single and single with one child, these tax increases are equal to approximately one-eighth the student’s total stipend income over four years. Interestingly, at higher income and tuition marriage status is the larger buffer, apparently due to a low initial tax burden for those with children at lower incomes. This also proves true for the CDTB of the average graduate student: Single = $18,451; single with one child = $14,941; MFJ = $13,325; MFJ with one child = $13,310 ( Figure 4C). Strikingly, the CDTB is always negative when tuition is not taxable, meaning that the new tax plans would reduce cumulative taxation when compared to the historical structure. These data indicate that graduate students would actually benefit from the House and Senate tax plans in the form of decreased tax burden, as long as tuition is not taxable.

**Figure 5.**
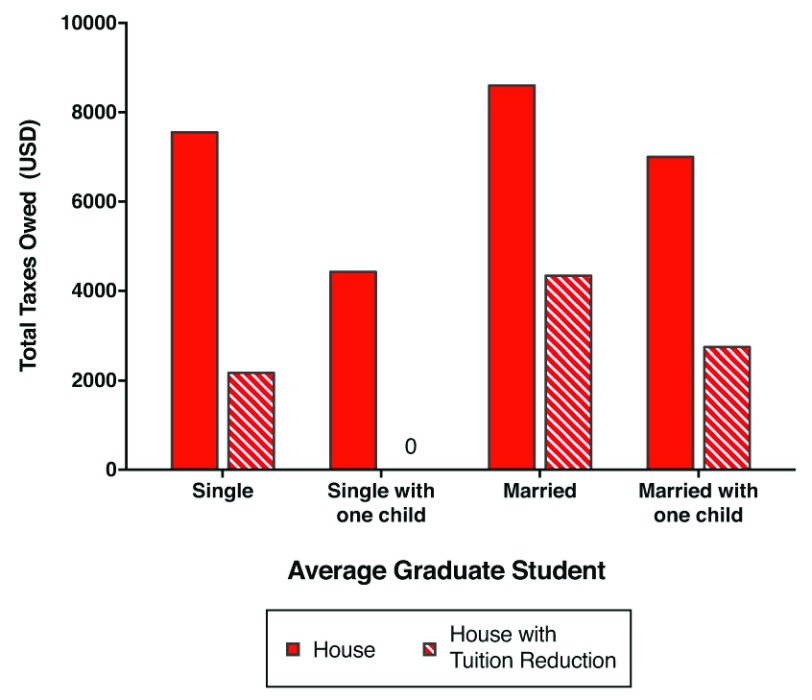
The effect of tuition reduction on graduate student taxation under the proposed House tax plan. Total Taxes Owed (TTO) in 2016, represented in USD, for an ‘average’ university graduate student filing either as single, single with one child, married (filing jointly), or married with one child (filing jointly). Data are coded according to the tax structure utilized to make each calculation ( Datasets 1–3, Tables S1–S6). These structures were the proposed House tax plan and the proposed House plan with Tuition Reduction. A “0” represents no taxes owed.

As has been alluded to throughout this work, it would appear that taxation of tuition is the single most important factor affecting graduate students in the House tax plan. In Figure 5, we show the effect of including tuition reduction in the House tax plan for one tax year, using 2016 estimations. For all graduate student family structures, changing this single factor immensely decreases the tax burden in all cases, anywhere from -$4,260 (MFJ with one child) to -$5,378 (single). This is a larger decrease than is realized for marriage (-$3,248 per person), having a child (-$3,125), or even the combination of these two (-$4,048 per person).

Calculation of 2013–2016 taxes for a public university graduate student using historical tax plans Click here for additional data file. Copyright: © 2018 Lawston PM and Parker MT 2018 Data associated with the article are available under the terms of the Creative Commons Zero "No rights reserved" data waiver (CC0 1.0 Public domain dedication).

Calculation of 2013–2016 taxes for a private university graduate student using historical tax plans Click here for additional data file. Copyright: © 2018 Lawston PM and Parker MT 2018 Data associated with the article are available under the terms of the Creative Commons Zero "No rights reserved" data waiver (CC0 1.0 Public domain dedication).

Calculation of 2013–2016 taxes for the ‘average’ university graduate student using historical tax plans Click here for additional data file. Copyright: © 2018 Lawston PM and Parker MT 2018 Data associated with the article are available under the terms of the Creative Commons Zero "No rights reserved" data waiver (CC0 1.0 Public domain dedication).

## Discussion

In this work, it was our goal to better understand the effects of the proposed Tax Cuts and Jobs Act on graduate students and to provide accurate information to help students, their supporters, and elected officials make informed decisions in their stand on the matter. Our findings indicate that taxable tuition would be the greatest contributor to graduate student tax burden across all four categories of filing status. However, when tuition is removed from the equation (whether in our modified House plan, the Senate plan, or by transition of students to sustaining fees) there is a decrease in tax burden in all situations. This gives validity to claims that these new tax plans would generally benefit low and middle-income families in the form of reduced taxation, at least in the short term ^23, 24^. But for graduate students, it would appear that these benefits would not be realized if tuition is considered taxable income.

Overall, we conclude that exclusion of tuition reduction from H.R.1, or for that matter any future iteration of U.S. Tax Code, would be an enormous financial burden on graduate students. What this would mean for graduate education in the US is uncertain, but will likely impact what schools will be able to host graduate programs, who can afford graduate education, and the diversity of the students within graduate programs.

## Data availability

The data referenced by this article are under copyright with the following copyright statement: Copyright: © 2018 Lawston PM and Parker MT

Data associated with the article are available under the terms of the Creative Commons Zero "No rights reserved" data waiver (CC0 1.0 Public domain dedication). 

Data for the two graduate student tuitions, stipends, and health waivers were provided directly from the authors from their personal tax information. These values can be found in the respective columns in Dataset 1 and Dataset 2.

Values used to calculate the ‘average’ graduate student were obtained via Glassdoor ^18^, MoneyUnder30 ^19^, and the American Academy of Sciences ^20^ (see Methods) or estimated from the mean of the two student values available in the absence of available data on health waivers and can be found in Dataset 3. All data is publicly available.

**Dataset 1.** Calculation of 2013–2016 taxes for a public university graduate student using historical tax plans. DOI, http://dx.doi.org/10.5256/f1000research.13385.d188156 ^25^

**Dataset 2.** Calculation of 2013–2016 taxes for a private university graduate student using historical tax plans. DOI, http://dx.doi.org/10.5256/f1000research.13385.d188157 ^26^

**Dataset 3.** Calculation of 2013–2016 taxes for the ‘average’ university graduate student using historical tax plans. DOI, http://dx.doi.org/10.5256/f1000research.13385.d188158 ^27^

## Supplementary material

**Table S1.** Calculation of 2013–2016 taxes for a public university graduate student using the proposed House tax plan, with and without tuition reduction.

**Table S2.** Calculation of 2013–2016 taxes for a private university graduate student using the proposed House tax plan, with and without tuition reduction.

**Table S3.** Calculation of 2013–2016 taxes for the ‘average’ university graduate student using the proposed House tax plan, with and without tuition reduction.

**Table S4.** Calculation of 2013–2016 taxes for a public university graduate student using the proposed Senate tax plan.

**Table S5.** Calculation of 2013–2016 taxes for a private university graduate student using the proposed Senate tax plan.

**Table S6.** Calculation of 2013–2016 taxes for the ‘average’ university graduate student using the proposed Senate tax plan.
