# Supplementary material for: Tuition reduction is the key factor determining tax burden of graduate students under the Tax Cuts and Job Act
Source: F1000Res. 2018 Feb 5;6:2166. Originally published 2017 Dec 21. [Version 2] doi: 10.12688/f1000research.13385.2 (PMC5806054; doi:10.12688/f1000research.13385.2)
Supplement: Peer review history

# Peer review: Referee response for version 2

**DOI:** 10.5256/f1000research.15152.r30657

**Published:** 2018-02-07

**Authors:** Elizabeth Garrett-Mayer

**Affiliations:** 1 Division Director, Biostatistics and Data Management, American Society of Clinical Oncology (ASCO), Alexandria, VA, USA

The revisions are OK.  I deem it acceptable for indexing.

I have read this submission. I believe that I have an appropriate level of expertise to confirm that it is of an acceptable scientific standard.

# Peer review: Referee response for version 1

**DOI:** 10.5256/f1000research.14533.r29283

**Published:** 2018-01-30

**Authors:** Elizabeth Garrett-Mayer

**Affiliations:** 1 Division Director, Biostatistics and Data Management, American Society of Clinical Oncology (ASCO), Alexandria, VA, USA

Page 3: What is TTO? Not defined prior to its introduction in the Methods. It is defined in the Results instead. 

 Assumptions regarding income, health waivers and stipends are not well-described. In table 1, what income level is this based on? In the results, it is stated that “this student” is on sustaining fees and not full tuition. How would one know that? The assumptions should be plainly described in the methods section AND in the caption of the table. It seems that the authors are actually following the tax burden of a single graduate student where each year (2013-2016) represents that students year in graduate school. This is not clear at all. The methods need to be substantially improved. 

 The increase to 386% is misleading. It would seem relevant to calculate change from the most recent year of data, not 2013. The “sustaining” status is not clearly defined. This is obviously critically important. In Table 1, why does 2015 have an asterisk in the top row but not other rows? It would seem more logical and less confusing to take a single year (e.g. 2016) and compare the tax implications for students in each of the different settings. That is, showing the 2016 tax burden under each plan for students with full tuition, sustaining fees, stipend, different levels of other income or filing status, etc. The way it is now, because the methods do not emphasize (nor do most of the figures), it looks like 2013 is very odd. 

 Why does that stipend double when filing jointly?

 There is no actual ‘data’, but the author provide the numbers on which they based their assumptions. I have serious concerns that the results will be misinterpreted due to the presentation of results.

I have read this submission. I believe that I have an appropriate level of expertise to confirm that it is of an acceptable scientific standard, however I have significant reservations, as outlined above.

# Response

**Published:** 2018-02-02

**Authors:** Michael Parker

**Affiliations:** Yale University, USA

Thank you for your time in reviewing our manuscript and for your insight and suggestions.  We address each comment and suggestion in the order presented and hope that the edits in Version 2 meet your expectations.

 The original comments are shown in bold type font and our responses in regular font. Where changes have been made to the manuscript, the relevant text has been copied and pasted here and is shown within quotation marks.

**Reviewer comment 1:**

**Page 3: What is TTO? Not defined prior to its introduction in the Methods. It is defined in the Results instead.**

 Authors response 1:

 We have added a paragraph devoted to TTO in the Methods section. It now reads as follows:

**“Total taxes owed (TTO)**: For each year 2013–2016, the appropriate tax brackets were applied to calculate the total taxes owed (TTO) based on stipends, health waivers, and tuition. Representation of TTO is calculated by year, and differences in TTO for each proposed tax structure are in relation to that same year’s historical tax structure TTO calculation. The income values used for these calculations as well as the numerous factors considered in tax calculations (i.e., column headers) can all be found in Datasets 1–3.”

**Reviewer comment 2a:**

**Assumptions regarding income, health waivers and stipends are not well-described.**

 Authors response 2a:

 To more fully describe the income, health waiver, and stipend, additional information was added to the new TTO paragraph in the Methods as cited above. For the single public and private university students, no assumptions are made; rather the self-reported data by each student is used as provided. Assumptions for the ‘average’ grad student are given under the title subtitle ‘Estimating the average graduate student’ in the Methods section.

**Reviewer comment 2b:**

**In table 1, what income level is this based on?**

 Authors response 2b:

 For the Historical, House w/tuition reduction, and Senate scenarios, the income level used to calculate the tax burden in Table 1 is the student stipend only. Under the House plan, the income level is the sum of the stipend, health waiver, and tuition amounts as all three would be considered taxable income. The supplementary tables, S1-S6, include the exact stipend, health waiver, and tuition amounts for each student and each year.

**Reviewer comment 2c:**

**In the results, it is stated that “this student” is on sustaining fees and not full tuition. How would one know that? The assumptions should be plainly described in the methods section AND in the caption of the table. It seems that the authors are actually following the tax burden of a single graduate student where each year (2013-2016) represents that students year in graduate school. This is not clear at all. The methods need to be substantially improved.**

Authors response 2c: An additional paragraph has been added to the methods section detailing the unique position of sustaining status. It reads as follows:

**“Sustaining fees**: Once graduate students progress from the class and teaching portions of their graduate work into the research intensive/exclusive portion, many schools no longer charge full tuition, but rather a smaller fee termed a “sustaining fee”. In the case of both graduate student salaries utilized in this work, this was the case, and the sustaining fees for each can be found in Datasets 1 and 2. The public student was on sustaining status in 2015 and 2016 while the private student was on sustaining status in 2016. The reduction in taxation is drastic when students transition from tuition status to sustaining status under plans that do not contain the tuition reduction provision (see Figures 1 and 2).”

               

 In addition, the introduction section has been revised to more clearly state that the years cited are in fact the progression of each graduate student through PhD programs:

 “To address these issues and to help graduate students and elected officials better inform themselves on the impact of proposed tax reform, we analyzed the historical taxes of two graduate students, one public university student and one private university student (the authors of this work), over the course of four years (2013-2016) in science PhD programs."

               

 Additionally, the caption of Table 1 had been revised as suggested to note the sustaining status and that the numbers can be found in the supplementary tables.

**Reviewer comment 3a:**

**The increase to 386% is misleading. It would seem relevant to calculate change from the most recent year of data, not 2013.**

Author response 3a: The ‘historical’ taxes are those that have been paid by each student in each year of graduate study (2013-2016), and serves essentially as the ‘control’ case. Thus, the differences (both absolute and percent change) use the ‘historical’ tax owed in the respective year as the reference value when calculating the changes under each plan. For example, the public student filing singly in 2013 owed $872, as the student’s stipend (and therefore gross income) was $18,724. If the House plan had been in effect at that time, the student’s gross income would have instead been the sum of the student’s stipend, health waiver ($1,273), and tuition waiver ($27,822), bringing the student’s gross income for 2013 to $47,819. Therefore, the student would have owed $4,238 in taxes in 2013, if the House plan had been in effect; an increase of 385% over the historical tax burden. We use this retrospective analysis approach to quantify the potential each of the proposed plans on graduate student tax burden.  

**Reviewer comment 3b:**

**The “sustaining” status is not clearly defined. This is obviously critically important. In Table 1, why does 2015 have an asterisk in the top row but not other rows?**

Authors’ response 3b:

 Thank you for calling the ambiguity related to sustaining fees to our attention, which was echoed by the second reviewer. As a result of these comments, we’ve revised the Methods section to include an additional paragraph which describes the sustaining fees/status. It now reads as follows:

 “Sustaining fees: Once graduate students progress from the class and teaching portions of their graduate work into the research intensive/exclusive portion, many schools no longer charge full tuition, but rather a smaller fee termed a “sustaining fee”. In the case of both graduate student salaries utilized in this work, this was the case, and the sustaining fees for each can be found in Datasets 1 and 2. The public student was on sustaining status in 2015 and 2016 while the private student was on sustaining status in 2016. The reduction in taxation is drastic when students transition from tuition status to sustaining status under plans that do not contain the tuition reduction provision (see Figures 1 and 2).”

 With respect to the asterisk in the top row, this was supposed to be included for the 2015 headers for the public student but erroneously excluded.  Thank you for bringing it to our attention. We have updated Table 1 to fix this issue.

**Reviewer comment 3c:**

**It would seem more logical and less confusing to take a single year (e.g. 2016) and compare the tax implications for students in each of the different settings. That is, showing the 2016 tax burden under each plan for students with full tuition, sustaining fees, stipend, different levels of other income or filing status, etc. The way it is now, because the methods do not emphasize (nor do most of the figures), it looks like 2013 is very odd.**

 We agree that presenting the data in the way suggested would more clearly display the impacts of each plan on the respective categories listed. However, a main goal of this work was to better understand the cumulative impacts of these potential plans over the course of a graduate student’s career; a career that is not typically not limited to any one of these settings (e.g., full tuition, sustaining, etc.) but rather is an aggregate of each of these and other factors that change yearly. Thus, we feel it provides a distinct advantage and is more representative to display the data in the way chosen. For example, Fig. 1 shows that despite the yearly variability in taxes owed, especially for the public university student (due to a 2016 increased stipend), the House plan greatly increases the tax burden for both students. However, the House plan with the tuition reduction maintained and the Senate plan both decrease the tax burden for both students.

**Reviewer comment 4:**

**Why does that stipend double when filing jointly?**

 Although the majority of graduate students are unmarried, we acknowledge that other family structures exist and these structures could be particularly vulnerable to drastic changes in the tax code. Thus, we extended the analysis to consider single graduate students with one child, married graduate students, and married graduate students with one child. As this analysis is centered specifically on graduate students, we consider only marriages where the couple is comprised of two graduate students at the same university. As a result, the income for the couple is defined as twice the individual student’s yearly stipend. We acknowledge that a married graduate student could be married to any variety of partners, and thus why we limit the analysis to only graduate student pairs.

 This is described in paragraph 5 of the results:

 “It is important to mention that in keeping with the subject of this study, namely, graduate students, married couple calculations assume that both partners are students at the same university, and therefore the income, tuition, and health waivers are double that of a single student."

**Reviewer comment 5:**

**There is no actual ‘data’, but the author provide the numbers on which they based their assumptions. I have serious concerns that the results will be misinterpreted due to the presentation of results.**

 Author response 5:

 The data provided are the actual student stipends, health waiver amounts, and tuition costs for four years for two graduate students – one at a public university and one at a private university. These data are taken directly from pay stubs and student financial statements willingly disclosed by the students; the authors of this manuscript. Although it would be ideal to have a large sample size of student financial information, we feel it is not necessary for the scope of this work. As public universities tend to offer lower stipends and private universities typically the highest, the public v. private university contrast offers lower and upper bounds on the potential impacts of each of these plans. This is confirmed by the information obtained from Glassdoor that shows the average stipend for a graduate student falls between that reported by the public and private university students.

 We hope that the explanations provided in these responses and the changes to the manuscript made as a result of the thoughtful reviewer comments have helped to clarify the data and assumptions and have generally improved the presentation of the manuscript.

# Peer review: Referee response for version 1

**DOI:** 10.5256/f1000research.14533.r29597

**Published:** 2018-01-23

**Authors:** Joshua D Hall

**Affiliations:** 1 Office of Graduate Education, University of North Carolina at Chapel Hill, Chapel Hill, NC, USA

This well-written article examines the financial impact that changes to the US tax code would have on graduate students who receive paid tuition as a benefit. This is a timely and important topic being discussed by graduate students and those involved with graduate education across the country. While the final, passed tax bill did not include taxation of graduate student tuition benefits, this study will provide useful data for demonstrating the significant financial burden such a change would levy upon graduate students if it comes up for discussion in the future.

 A few specific comments and suggestions.

1. As a technical point, the term “tuition reduction” is used to describe the benefit that graduate students would potentially no longer be able to deduct. However, in biomedical graduate programs that I am familiar with, the grad student tuition is not waived or reduced, but paid on behalf of the graduate student by their department or thesis lab. This may be what the authors meant by this term, but this should be clarified to avoid confusion.
2. For estimation of “average” graduate student salaries, it is unclear if this is for graduate students of all disciplines or for students from only STEM disciplines, which tend to have higher stipend levels. The $30,603 average seems high for an average inclusive of all graduate students independent of discipline.
3. Please define “TTO” the first time it is used.
4. I’m not familiar with the term “sustaining fees”. I’m deducing that this means the student did not have to pay tuition for that year for some reason, but this should be defined explicitly in the text, especially because it has a big influence on the results in Table/Figure 1.
5. While individual funding structures for graduate students can vary widely from discipline to discipline (and even year to year as the data from Fig 1 indicates for individual students), the analysis in Figure 3 is particularly useful for showing the taxation impact on graduate students overall as a result of a taxation plan that includes taxation of tuition benefits.

I have read this submission. I believe that I have an appropriate level of expertise to confirm that it is of an acceptable scientific standard.

# Response

**Published:** 2018-02-02

**Authors:** Michael Parker

**Affiliations:** Yale University, USA

Thank you for your time in reviewing our manuscript and for your insight and suggestions.  We address each by the number system you utilized for commenting and hope that the edits in Version 2 meet your expectations.

**Reviewer comment 1:**

**As a technical point, the term “tuition reduction” is used to describe the benefit that graduate students would potentially no longer be able to deduct. However, in biomedical graduate programs that I am familiar with, the grad student tuition is not waived or reduced, but paid on behalf of the graduate student by their department or thesis lab. This may be what the authors meant by this term, but this should be clarified to avoid confusion.**

Authors response 1.

Thanks for pointing this out. We edited the wording in the introduction to make it clearer that this typically refers to tuition paid for the student by department money. This section now reads:

“However, the most jarring change for graduate students is the removal of tuition reduction ^2^ , the provision in current tax code allowing students to exclude money provided by their graduate program to cover their tuition costs from their taxable income.”

**Reviewer comment 2:**

**For estimation of “average” graduate student salaries, it is unclear if this is for graduate students of all disciplines or for students from only STEM disciplines, which tend to have higher stipend levels. The $30,603 average seems high for an average inclusive of all graduate students independent of discipline.**

Authors response 2:

Although we agree that this average seems high, it is the best approximation we can make from anonymous self-reporting. It would appear that the Glassdoor site may have a bias toward STEM fields, but it is not clear. We provided a statement in the methods to note this uncertainty, which hopefully provides more clarity on the nature of the approximation. This revised section reads as follows:

“We estimated the ‘average’ stipend using the current Glassdoor approximation of $30,603 ^10^ and then back-calculated for 2013–2015 assuming a 2% yearly increase, as was observed in the public and private student stipends. One consideration in this approximation is that these data are self-reported and not field specified and since STEM fields typically have higher stipends than humanities, bias in either discipline’s direction may skew this value.”

**Reviewer comment 3:**

**Please define “TTO” the first time it is used.**

Authors’ response 3:

Done. Additionally, we have added an additional section describing TTO more fully to the methods to address both reviewer comments about TTO.

**Reviewer comment 4:**

**I’m not familiar with the term “sustaining fees”. I’m deducing that this means the student did not have to pay tuition for that year for some reason, but this should be defined explicitly in the text, especially because it has a big influence on the results in Table/Figure 1.**

Authors’ response 4:

Thanks for calling this to our attention. Students are typically placed on sustaining status after they have completed their coursework, but still have other degree requirements to fill. This is most often research and/or the completion of a thesis. Tuition is not charged since the student is not enrolled in classes. Instead a sustaining fee is charged in exchange for the student’s active status and to allow continued use of the University resources. We have revised the Methods section to include an additional paragraph which describes the sustaining fees/status. It now reads as follows:

“Sustaining fees: Once graduate students progress from the class and teaching portions of their graduate work into the research intensive/exclusive portion, many schools no longer charge full tuition, but rather a smaller fee termed a “sustaining fee”. In the case of both graduate student salaries utilized in this work, this was the case, and the sustaining fees for each can be found in Datasets 1 and 2. The public student was on sustaining status in 2015 and 2016 while the private student was on sustaining status in 2016. The reduction in taxation is drastic when students transition from tuition status to sustaining status under plans that do not contain the tuition reduction provision (see Figures 1 and 2).”

**Reviewer comment 5:**

**While individual funding structures for graduate students can vary widely from discipline to discipline (and even year to year as the data from Fig 1 indicates for individual students), the analysis in Figure 3 is particularly useful for showing the taxation impact on graduate students overall as a result of a taxation plan that includes taxation of tuition benefits.**

Authors’ response 5:

Thank you for your kind words and for your support of the reasoning behind this figure.  We, too, believe that these data are important and hope they will be widely useful for readers of our manuscript.
